# DABCO Catalyzed Synthesis of Xanthene Derivatives in Aqueous Media

**DOI:** 10.1155/2013/526173

**Published:** 2013-03-18

**Authors:** Pradeep Paliwal, Srinivasa Rao Jetti, Anjna Bhatewara, Tanuja Kadre, Shubha Jain

**Affiliations:** Laboratory of Heterocycles, School of Studies in Chemistry & Biochemistry, Vikram University, Ujjain, Madhya Pradesh 456010, India

## Abstract

The reaction of 5,5-dimethylcyclohexane-1,3-dione with various heteroarylaldehydes afforded the corresponding heteroaryl substituted xanthene derivatives **1(a–f)**. The reaction proceeds via the initial Knoevenagel, subsequent Michael, and final heterocyclization reactions using 1,4-diazabicyclo[2.2.2]octane (DABCO) as a catalyst in aqueous media. The synthesized heteroaryl substituted xanthenes **1(a–f)** reacted with malononitrile to obtain different alkylidenes **2(a–f)**. Short reaction time, environmentally friendly procedure, avoiding of cumbersome apparatus, and excellent yields are the main advantages of this procedure which makes it more economic than the other conventional methods.

## 1. Introduction

In the past few decades, the synthesis of new heterocyclic compounds has been a subject of great interest due to their wide applicability. The importance of multicomponent reactions in organic synthesis has been recognized, and considerable efforts have been focused on the design and development of one-pot procedures for the generation of libraries of heterocyclic compounds [1, 2]. Multicomponent reactions (MCRs) have emerged as an important tool for building of diverse and complex organic molecules through carbon-carbon and carbon-heteroatom bond formations taking place in tandem manner [3]. Particularly, in the last three decades a number of three- and four-component reactions have been developed [4–6].

**Scheme 1:**
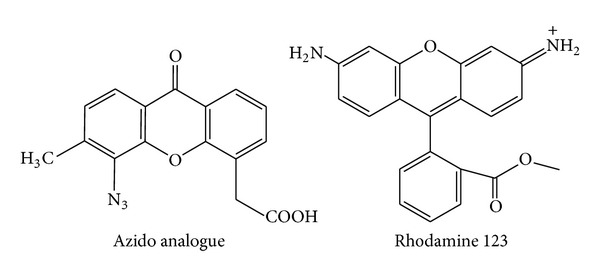


Xanthene derivatives are very important heterocyclic compounds and have been widely used as dyes [7] and fluorescent materials for visualization of biomolecules and in laser technologies [8]. They have also been reported for their agricultural bactericide activity [9] and anti-inflammatory [10] and antiviral activity [11]. These compounds are also utilized as antagonists for paralyzing action of zoxazolamine and in photodynamic therapy [12]. Due to their wide range of applications, these compounds have received a great deal of attention in connection with their synthesis. A wide variety of methods for the preparation of the xanthenes have been reported [13–19]. However, many of these methods are associated with several shortcomings such as long reaction times (16 h to 5 days), expensive reagents, harsh conditions, low product yields, and use of toxic organic solvents. Diazabicyclo[2.2.2]octane (DABCO) is an inexpensive, nontoxic, and commercially available catalyst that can be used in laboratory without special precautions [20–22]. But, it has not been used as a catalyst in xanthene synthesis; only a few reports are therein the literature [23–25]. This prompted us to develop a new synthetic method for heteroaryl substituted xanthenes using DABCO as a catalyst (see Scheme 1).

**Scheme 2:**
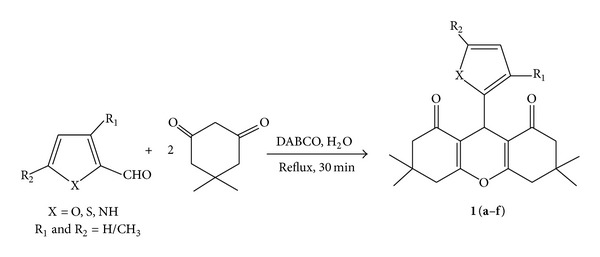


With our continued interest in the synthesis of heterocyclic systems [26] and application of DABCO as a catalyst in organic synthesis [27] herein, we wish to report a facile condensation of heteroarylaldehyde, 5,5′-dimethyl-1,3-cyclohexanedione (dimedone), in the presence of catalytic amount of DABCO to produce a variety of 1,8-dioxo-octahydroxanthenes derivatives **1(a–f)** (Scheme 2).

## 2. Results and Discussion

In order to optimize the reaction conditions, the synthesis of compound **1d** was used as a model reaction. Therefore, a mixture of 3-methyl thienaldehyde (1 mmol), 5,5-dimethyl cyclohexane-1,3-dione (2 mmol) in H_2_O was refluxed for an appropriate time as indicated by TLC using different amounts of DABCO (Table 1). The efficiency of the reaction is mainly affected by the amount of the catalyst. Traces of the product could be detected in the absence of this catalyst (entry **1**), while good results were obtained in the presence of DABCO. The optimal amount of the catalyst was 10 mmol% (entry **6**); the higher amount of the catalyst did not increase the yield noticeably (entry **7**).

**Scheme 3:**
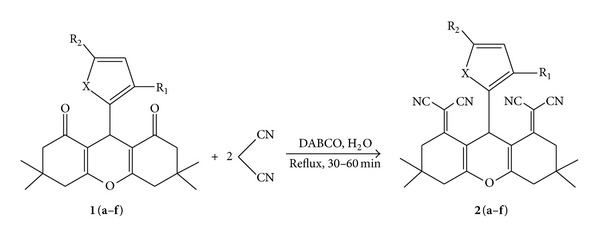


The synthesized products **1(a–f)** in Scheme 2 were further treated with malononitrile to obtain corresponding alkylidenes **2(a–f)** by the Knoevenagel reaction. The reaction involves the attack of malononitrile on two carbonyl groups (C=O) of xanthene derivatives to form alkylidene malononitrile within 60 min. using DABCO as an organic catalyst (Scheme 3).

In order to extend the range of substrates, we employed a wide range of aldehydes in the presence of 10 mmol% DABCO under similar conditions. It was found that this method is effective with a variety of substituted heteroarylaldehydes independent of the nature of the substituent on the heteroaromatic ring and obtained satisfactory results (Table 2).

**Scheme 4:**
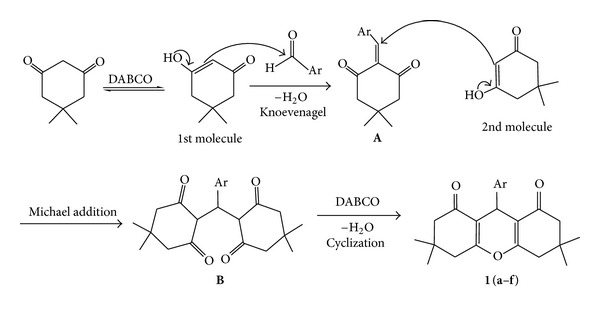


The formation of the products **1(a–f)** was assumed to proceed via formation of a Knoevenagel product which on addition of 2nd molecule to give the Michael adduct intermediate was followed by cyclization reaction (Scheme 4). An *α*,*α*′-bis(arylidene)cycloalkanone **A **was first condensed with dimedone to afford the **B** on addition of 2nd molecule of dimedone; this step can be regarded as a Michael addition reaction. The intermediate **B** was cyclized by nucleophilic attack of the OH group on the C=C moiety and gave the expected products **1(a–f)**.

## 3. Conclusion

In summary, we have reported an efficient, simple, convenient, and straightforward practical one-pot procedure for the synthesis of **1(a–f)** in aqueous media. Reaction of malononitrile on the synthesized products **1(a–f)** gave corresponding alkylidene derivatives **2(a–f)** in good yields. All starting materials are readily available from commercial sources. Moreover, there is no need for dry solvents or protecting gas atmospheres. Using DABCO as a catalyst offers advantages including simplicity of operation, easy workup, time minimizing, and high yields of products. The procedure is very simple and can be used as an alternative to the existing procedures.

## 4. Experimental

### 4.1. General

The chemicals used in the synthesis of the octahydroxanthene-1,8-diones were obtained from the Merck and Aldrich Chemical Co. All chemicals and solvents used for the synthesis were of analytical reagent grade. Reactions were monitored by thin layer chromatography on 0.2 mm silica gel F-252 (Merck) plates. Melting points were determined by open capillary method and were uncorrected.^ 1^H (400 MHz) and ^13^C (200 MHz) spectra were recorded on Bruker 3000 NMR spectrometer in CDCl_3_/DMSO-*d *_6_ (with TMS for ^1^H and CDCl_3_ as internal references) unless otherwise specified stated.

### 4.2. General Procedure for the Synthesis of Heteroaryl Substituted Xanthenes ** 1(a–f)**

A mixture of 5-membered, heteroarylaldehyde (1 mmol), 5,5-dimethylcyclohexane-1,3-dione (2 mmol), and DABCO (10 mmol%) in H_2_O (20 mL) was refluxed for 30 min. The progress of the reaction was monitored by TLC. After completion of the reaction, the mixture was cooled to room temperature, and the solid was filtered off and washed with H_2_O. The crude product was purified by recrystallization from 95% ethanol.

### 4.3. General Procedure for the Synthesis of Alkylidenes ** 2(a–f)**

A mixture of *heteroaryl substituted xanthenes* (1 mmol), malononitrile (2 mmol), and DABCO (10 mmol %) in H_2_O (20 mL) was stirred for 60 min. The progress of the reaction was monitored by TLC. After completion of the reaction, the mixture was cooled to room temperature and the solid was filtered off and washed with H_2_O. The crude product was purified by column chromatographic technique using hexane: ethyl acetate.

### 4.4. Spectral Data of Compounds

*9-(Furan-2-yl)-3,3,6,6-tetramethyl-3,4,5,6,7,9-hexahydro-1H-xanthene-1,8(2H)-dione* **(1a)**. ^1^H NMR (400 MHz, CDCl_3_) *δ*: 1.014 (s, 6H, 2×CH_3_), 1.084 (s, 6H, 2 × CH_3_), 2.235 (s, 4H, 2 × CH_2_), 2.425 (s, 4H, CH_2_), 4.941 (s, 1H, CH), 6.159–6.181 (m, 2H, Ar-H), 7.133–7.139 (d, 1H, Ar-H); IR *ν*: 3078 cm^−1^ (Ar-H), 2865 cm^−1^ (Aliph. C–H), 1730 cm^−1^ and 1673 cm^−1^ (C=O), 1602 cm^−1^ (C=C), 1180 cm^−1^ (C–O–C). Anal. calcd for C_21_H_24_O_4_: C 74.09, H 7.11; found C 74.03, H 7.07.

*3,3,6,6-Tetramethyl-9-(5-methylfuran-2-yl)-3,4,5,6,7,9-hexahydro-1H-xanthene-1,8(2H)-dione* **(1b)**. ^1^H NMR (400 MHz, CDCl_3_) *δ*: 1.039 (s, 6H, 2 × CH_3_), 1.109 (s, 6H, 2 × CH_3_), 2.109 (s, 4H, 2 × CH_2_), 2.551 (s, 4H, CH_2_), 4.832 (s, 1H, CH), 6.108–6.226 (m, 2H, Ar-H), 3.228 (s, 3H, Ar-CH_3_); IR *ν*: 3109 cm^−1^ (Ar-H), 2905 cm^−1^ (Alih. C–H), 1722 cm^−1^ and 1688 cm^−1^ (C=O), 1630 cm^−1^ (C=C), 1172 cm^−1^ (C–O–C). Anal. calcd for C_22_H_26_O_4_: C 74.55, H 7.39; found C 75.28, H 6.88.

*3,3,6,6-Tetramethyl-9-(thiophen-2-yl)-3,4,5,6,7,9-hexahydro-1H-xanthene-1,8(2H)-dione* **(1c)**. ^1^H NMR (400 MHz, CDCl_3_) *δ*: 1.208 (s, 12H, 4 × CH_3_), 2.109 (s, 4H, 2 × CH_2_), 2.401 (s, 4H, 2 × CH_2_), 4.622 (s, 1H, CH), 6.554 (d, 1H, Ar-H), 6.828 (d, 1H, Ar-H), 7.298 (dd, 1H, Ar-H); IR *ν*: 3135 (Ar-H), 2920 cm^−1^ (Aliph. C–H), 1716 cm^−1^ (C=O), 1648 cm^−1^ and 1620 cm^−1^ (C=C), 1108 cm^−1^ (C–O–C). Anal. calcd for C_21_H_24_O_3_S: C 70.75, H 6.79, S 8.99; found C 71.33, H 6.28, S 8.49.

*3,3,6,6-Tetramethyl-9-(3-methylthiophen-2-yl)-3,4,5,6,7,9-hexahydro-1H-xanthene-1,8(2H)-dione* **(1d)**. ^1^H NMR (400 MHz, CDCl_3_) *δ*: 1.100 (s, 12H, 4 × CH_3_), 3.035 (s, 3H, Ar-CH_3_), 2.281 (s, 4H, 2 × CH_2_), 2.544 (s, 4H, 2 × CH_2_), 4.875 (s, 1H, CH), 6.478 (d, 1H, Ar-H), 6.824 (d, 1H, Ar-H); IR *ν*: 3042 cm^−1^ (Ar-H), 2963 cm^−1^ (C–H), 1730 cm^−1^ (C=O), 1607 cm^−1^ and 1588 cm^−1^ (C=C), 1150 cm^−1^ (C–O–C). Anal. calcd for C_22_H_26_O_3_S: C 71.32, H 7.07, S 8.65; found C 71.28, H 7.09, S 8.69.

*3,3,6,6-Tetramethyl-9-(5-methylthiophen-2-yl)-3,4,5,6,7,9-hexahydro-1H-xanthene-1,8(2H)-dione* **(1e)**. ^1^H NMR (400 MHz, CDCl_3_) *δ*: 1.288 (s, 12H, 4 × CH_3_), 2.988 (s, 3H, Ar-CH_3_), 2.448 (s, 4H, 2 × CH_2_), 2.722 (s, 4H, 2 × CH_2_), 4.658 (s, 1H, CH), 6.234 (d, 1H, Ar-H), 6.775 (d, 1H, Ar-H); IR *ν*: 3090 cm^−1^ (Ar-H), 2882 cm^−1^ (Aliph. C–H), 1716 cm^−1^ (C=O), 1632 cm^−1^ and 1610 cm^−1^ (C=C), 1148 cm^−1^ (C–O–C). Anal. calcd for C_22_H_26_O_3_S: C 71.32, H 7.07, S 8.65; found C 71.54, H 7.68, S 9.14.

*3,3,6,6-Tetramethyl-9-(1H-pyrrol-2-yl)-3,4,5,6,7,9-hexahydro-1H-xanthene-1,8(2H)-dione* **(1f)**. ^1^H NMR (400 MHz, CDCl_3_) *δ*: 1.018–1.146 (m, 12H, 4 × CH_3_), 2.154 (br s, 8H, 4 × CH_2_, 5.601 (s, 1H, CH), 6.957–6.970 (s, 1H, Ar-H), 6.698–6.710 (d, 1H, Ar-H), 6.162 (dd, 1H, Ar-H), 9.570 (br s, 1H, NH); IR *ν*: 3397 cm^−1^, 3328 cm^−1^ (N–H), 3065 cm^−1^ (Ar-H), 2978v (Aliph. C–H), 1680 cm^−1^ (C=O), 1604 cm^−1^ and 1469 cm^−1^ (C=C), 1145 cm^−1^ (COC). Anal. calcd for C_21_H_25_NO_3_: C 74.31, H 7.42, N 4.13; found C 74.26, H 7.46, N 4.15. 

*2,2 *′*-(3,3,6,6-Tetramethyl-9-(furan-2-yl)-3,4,5,6,7,9-hexahydro-1H-xanthene-1,8(2H)-diylidene)dimalononitrile* **(2a**). ^1^H NMR (400 MHz, CDCl_3_) *δ*: 1.016 (s, 6H, 2 × CH_3_), 1.128 (s, 6H, 2 × CH_3_), 2.246 (s, 4H, 2 × CH_2_), 2.665 (s, 4H, CH_2_), 4.941 (s, 1H, CH), 6.154–6.188 (m, 2H, Ar-H), 7.138–7.144 (d, 1H, Ar-H); IR *ν*: 3078 cm^−1^ (Ar-H), 2865 cm^−1^ (aliph. C–H), 2224 cm^−1^ (CN), 1716 cm^−1^ and 1684 cm^−1^ (C=O), 1620 cm^−1^ (C=C), 1154 cm^−1^ (C–O–C). Anal. calcd for C_27_H_24_N_4_O_2_: C 74.29, H 5.54, N 12.84; found C 73.82, H 5.69, N 12.09.

*2,2 *′*-(3,3,6,6-Tetramethyl-9-(5-methylfuran-2-yl)-3,4,5,6,7,9-hexahydro-1H-xanthene-1,8(2H)-diylidene)dimalononitrile* **(2b)**. ^1^H NMR (400 MHz, CDCl_3_) *δ*: 0.986 (s, 6H, 2 × CH_3_), 1.235 (s, 6H, 2 × CH_3_), 2.244 (s, 4H, 2 × CH_2_), 2.658 (s, 4H, CH_2_), 4.988 (s, 1H, CH), 6.159–6.181 (m, 2H, Ar-H), 3.286 (s, 3H, Ar-CH_3_); IR *ν*: 3058 cm^−1^ (Ar-H), 2944 cm^−1^ (Aliph. C–H), 2224 cm^−1^ (CN), 1714 cm^−1^ and 1682 cm^−1^ (C=O), 1622 cm^−1^ (C=C), 1144 cm^−1^ (C–O–C). Anal. calcd for C_28_H_26_N_4_O_2_: C 74.65, H 5.82, N 12.44; found C 75.11, H 6.08, N 11.88.

*2,2 *′*-(3,3,6,6-Tetramethyl-9-(thiophen-2-yl)-3,4,5,6,7,9-hexahydro-1H-xanthene-1,8(2H)-diylidene)dimalononitrile* **(2c)**. ^1^H NMR (400 MHz, CDCl_3_) *δ*: 1.029 (s, 6H, 2 × CH_3_), 1.208 (s, 6H, 2 × CH_3_), 2.248 (s, 4H, 2 × CH_2_), 2.659 (s, 4H, CH_2_), 4.745 (s, 1H, CH), 6.686–6.789 (m, 2H, Ar-H), 7.252–7.263 (d, 1H, Ar-H); IR *ν*: 3078 cm^−1^ (Ar-H), 2988 cm^−1^ (Aliph. C–H), 2224 cm^−1^ (CN), 1710 cm^−1^ and 1688 cm^−1^ (C=O), 1626 cm^−1^ (C=C), 1164 cm^−1^ (C–O–C). Anal. calcd for C_27_H_24_N_4_OS: C 71.65, H 5.35, N 12.84, S 7.09; found C 71.18, H 5.74, N 12.12, S 7.83.

*2,2 *′*-(3,3,6,6-Tetramethyl-9-(3-methylthiophen-2-yl)- 3,4,5,6,7,9-hexahydro-1H-xanthene-1,8(2H)-diylidene)dimalononitrile* **(2d)**. ^1^H NMR (400 MHz, CDCl_3_) *δ*: 1.044 (s, 6H, 2 × CH_3_), 1.301 (s, 6H, 2 × CH_3_), 2.144 (s, 4H, 2 × CH_2_), 2.656 (s, 4H, CH_2_), 4.886 (s, 1H, CH), 6.136–6.172 (m, 2H, Ar-H), 3.114 (s, 3H, Ar-CH_3_); IR *ν*: 3098 cm^−1^ (Ar-H), 2898 cm^−1^ (Aliph. C–H), 2224 cm^−1^ (CN), 1710 cm^−1^ and 1682 cm^−1^ (C=O), 1663 cm^−1^ (C=C), 1156 cm^−1^ (C–O–C). Anal. calcd for C_28_H_26_N_4_OS: C 72.07, H 5.62, N 12.01, S 6.87; found C 71.12, H 5.28, N 12.84, S 7.15.

*2,2 *′*-(3,3,6,6-Tetramethyl-9-(5-methylthiophen-2-yl)- 3,4,5,6,7,9-hexahydro-1H-xanthene-1,8(2H)-diylidene)dimalononitrile* **(2e)**. ^1^H NMR (400 MHz, CDCl_3_) *δ*: 1.022 (s, 6H, 2 × CH_3_), 1.308 (s, 6H, 2 × CH_3_), 2.224 (s, 4H, 2 × CH_2_), 2.538 (s, 4H, CH_2_), 4.908 (s, 1H, CH), 6.108–6.191 (m, 2H, Ar-H), 3.257 (s, 3H, Ar-CH_3_); IR *ν*: 3086 cm^−1^ (Ar-H), 2910 cm^−1^ (Aliph. C–H), 2224 cm^−1^ (CN), 1728 cm^−1^ and 1692 cm^−1^ (C=O), 1605 cm^−1^ (C=C), 1162 cm^−1^ (C–O–C). Anal. calcd for C_28_H_26_N_4_OS: C 72.07, H 5.62, N 12.01, S 6.87; found C 71.43, H 5.12, N 12.77, S 7.25.

*2,2 *′*-(3,3,6,6-Tetramethyl-9-(pyrrol-2-yl)-3,4,5,6,7,9-hexahydro-1H-xanthene-1,8(2H)-diylidene)dimalononitrile* **(2f)**. ^1^H NMR (400 MHz, CDCl_3_) *δ*: 1.022 (s, 6H, 2 × CH_3_), 1.063(s, 6H, 2 × CH_3_), 2.268 (s, 4H, 2 × CH_2_), 2.569 (s, 4H, CH_2_), 4.858 (s, 1H, CH), 6.168–6.198 (m, 2H, Ar-H), 7.124–7.138 (d, 1H, Ar-H), 8.986 (br s, 1H, NH); IR *ν*: 3064 cm^−1^ (Ar-H), 2936 cm^−1^ (Aliph. C–H), 2224 cm^−1^ (CN), 1710 cm^−1^ and 1678 cm^−1^ (C=O), 1619 cm^−1^ (C=C), 1166 cm^−1^ (COC). Anal. calcd for C_21_H_24_O_4_: C 74.46, H 5.79; found C 74.12, H 6.08.

## Figures and Tables

**Table 1:** Influence of the amounts of DABCO on the synthesis of **1d** at reflux temperature^a^.

Entry	Catalyst	Amount of catalyst	Time (min)	Yield^b^ (%)
		(mmol%)		
1	None	—	80	Trace
2	DABCO	1	70	67
3	DABCO	2	60	74
4	DABCO	3	50	82
5	DABCO	5	40	89
6	DABCO	10	30	96
7	DABCO	15	30	96

^a^Reaction conditions: 3-methyl thienaldehyde (1 mmol), dimedone (2 mmol) in water (20 mL) under reflux temperature. ^b^Isolated yields.

**Table 2:** Synthesis of heteroaryl substituted xanthenes and its alkylidene derivatives^a,b^.

Entry	X	R_1_	R_2_	Time (min)	Product	Yield (%)^c^	M.P (°C)
1	O	H	H	30	**1a**	94	168-169
2	O	H	CH_3_	30	**1b**	92	158–160
3	S	H	H	30	**1c**	95	142–144
4	S	CH_3_	H	30	**1d**	96	156-157
5	S	H	CH_3_	30	**1e**	94	145–147
6	NH	H	H	30	**1f**	87	88–90
7	O	H	H	60	**2a**	78	212-213
8	O	H	CH_3_	60	**2b**	76	183–185
9	S	H	H	60	**2c**	81	197-198
10	S	CH_3_	H	60	**2d**	77	170–172
11	S	H	CH_3_	60	**2e**	83	177–179
12	NH	H	H	60	**2f**	87	112–114

^a^Reaction conditions: heteroarylaldehyde (1 mmol), dimedone (2 mmol), and DABCO (10 mmol%) in water (20 mL) under reflux temperature. ^b^Reaction conditions: **1a–f** (1 mmol), malononitrile (2 mmol), and DABCO (10 mmol%) in water (20 mL) under reflux temperature. ^c^Isolated yields.
